# Regulation of sugar metabolism genes in the nitrogen-dependent susceptibility of tomato stems to *Botrytis cinerea*

**DOI:** 10.1093/aob/mcaa155

**Published:** 2020-08-27

**Authors:** Nathalie Lacrampe, Félicie Lopez-Lauri, Raphaël Lugan, Sophie Colombié, Jérôme Olivares, Philippe C Nicot, François Lecompte

**Affiliations:** 1 PSH unit, INRAE, Avignon, France; 2 UMR Qualisud, Avignon Université, Avignon, France; 3 UMR 1332 BFP, INRAE, Univ Bordeaux, Villenave d’Ornon, France; 4 Plant pathology unit, INRAE, Montfavet, France

**Keywords:** *Botrytis cinerea*, fructokinase, hexokinase, invertase, nitrate, plant pathogen interaction, *Solanum lycopersicum* (tomato), sucrose synthase

## Abstract

**Background and Aims:**

The main soluble sugars are important components of plant defence against pathogens, but the underlying mechanisms are unclear. Upon infection by *Botrytis cinerea*, the activation of several sugar transporters, from both plant and fungus, illustrates the struggle for carbon resources. In sink tissues, the metabolic use of the sugars mobilized in the synthesis of defence compounds or antifungal barriers is not fully understood.

**Methods:**

In this study, the nitrogen-dependent variation of tomato stem susceptibility to *B. cinerea* was used to examine, before and throughout the course of infection, the transcriptional activity of enzymes involved in sugar metabolism. Under different nitrate nutrition regimes, the expression of genes that encode the enzymes of sugar metabolism (invertases, sucrose synthases, hexokinases, fructokinases and phosphofructokinases) was determined and sugar contents were measured before inoculation and in asymptomatic tissues surrounding the lesions after inoculation.

**Key Results:**

At high nitrogen availability, decreased susceptibility was associated with the overexpression of several genes 2 d after inoculation: sucrose synthases *Sl-SUS1* and *Sl-SUS3*, cell wall invertases *Sl-LIN5* to *Sl-LIN9* and some fructokinase and phosphofructokinase genes. By contrast, increased susceptibility corresponded to the early repression of several genes that encode cell wall invertase and sucrose synthase. The course of sugar contents was coherent with gene expression.

**Conclusions:**

The activation of specific genes that encode sucrose synthase is required for enhanced defence. Since the overexpression of fructokinase is also associated with reduced susceptibility, it can be hypothesized that supplementary sucrose cleavage by sucrose synthases is dedicated to the production of cell wall components from UDP-glucose, or to the additional implication of fructose in the synthesis of antimicrobial compounds, or both.

## INTRODUCTION

The leaves of healthy plants synthesize carbohydrates through photosynthesis and they export sucrose, which is carried via the phloem and unloaded in sink tissues such as stems, roots or fruit via sucrose transporters (SWEET transporters) or plasmodesmata (Ruan, 2014). Once inside the parenchyma of sink tissues, the sucrose can enter the cells via transporters or be cleaved in the apoplast by cell wall invertases (EC 3.2.1.26) into glucose and fructose, which are then imported into the cytoplasm via hexose transporters (sugar transporter proteins). Cytosolic sucrose can be stored in vacuoles and eventually cleaved into glucose and fructose by a vacuolar invertase. Cytosolic sucrose can also be reversibly cleaved by sucrose synthases (EC 2.4.1.13), thus yielding fructose and UDP-glucose, the latter being a precursor of cell wall polysaccharides (Proels and Hückelhoven, 2014; Stein and Granot, 2019). Glucose and fructose enter glycolysis after phosphorylation by hexokinases (EC 2.7.1.1) or fructokinases (EC 2.7.1.4). Hexokinases are the only known enzymes able to phosphorylate glucose, but they can also phosphorylate fructose, though with lower affinity (Pego and Smeekens, 2000; Granot, 2007; Aguilera-Alvarado and Sanchez-Nieto, 2017; Stein and Granot, 2018). In the next step of glycolysis, glucose-6-phosphate is converted into fructose-6-phosphate, which is phosphorylated by phosphofructokinases (EC 2.7.1.11) into fructose-1,6-bisphosphate.

Most of the genes that encode these enzymes have already been described in tomato. *Sl-LIN5* to *Sl-LIN9* code for cell wall invertases, *Sl-VI* codes for a vacuolar invertase and *Sl-NI* for a mitochondrial invertase. *Sl-SUS1* and *Sl-SUS3* to *Sl-SUS7* encode six sucrose synthase isozymes. Among the hexokinase genes *Sl-HXK1* to *Sl-HXK6*, three are located in the cytosol and associated with the mitochondrial membrane (*Sl-HXK1* to *Sl-HXK3*), one is plastidial (*Sl-HXK4*) and two have not been located (*Sl-HXK5* and *Sl-HXK6*). Among the four fructokinases reported in the literature, three are cytosolic (*Sl-FRK1*, *Sl-FRK2* and *Sl-FRK4*) and one is plastidial (*Sl-FRK3*). In addition, eight putative phosphofructokinases have been identified in the tomato genome (*Sl-PFK1* to *Sl-PFK8*).

Host sugars play a central role in plant–pathogen interactions (Rojas *et al.*, 2014). The main soluble sugars, such as sucrose, glucose and fructose, are thought to be involved in both the production and the regulation of antifungal metabolites and the maintenance of cell homeostasis, and they can also be used as a substrate for pathogen growth (Morkunas and Ratajczak, 2014). During pathogen infection, host metabolism is substantially altered. The pathogen colonization of plant source tissues induces an inhibition of photosynthesis along with an activation of respiratory processes such as glycolysis, the TCA cycle and the mitochondrial electron transport chain, which are required for defence (Berger *et al.*, 2007; Kanwar and Jha, 2019). The host cell machinery is manipulated by many pathogens for nutrient release (Fatima and Senthil-Kumar, 2015; Oliva and Quibod, 2017). Biotic stresses have been found to increase cell wall invertase activity (Berger *et al.*, 2007; Bolton, 2009; Proels and Hückelhoven, 2014) and to alter sucrose synthase at different levels: gene expression in leaf veins and syncytia (Hren *et al.*, 2009; Cabello *et al.*, 2014), the protein level in the phloem (Brzin *et al.*, 2011) and enzymatic activity in the wheat spikelet (Rios *et al.*, 2017).

*Botrytis cinerea* is a plant pathogenic fungus with a broad host range. Unlike biotrophs, necrotrophic pathogens such as *B. cinerea* kill host cells and feed on decayed tissues (Glazebrook, 2005). The metabolic adaptation of the host to infection by necrotrophic fungi has not fully been elucidated. An inhibition of photosynthesis activity measured using chlorophyll fluorescence was observed in tomato leaves infected by *B. cinerea* (Berger *et al.*, 2004). Gene transcripts linked to photosynthesis activity were also repressed in *Arabidopsis* leaves (Windram *et al.*, 2012), lettuce leaves (De Cremer *et al.*, 2013) and immature grape berries (Agudelo-Romero *et al.*, 2015). However, an increased photosynthetic rate was also observed in the area surrounding the infection site of tomato leaves (Berger *et al.*, 2004). In addition, sugar transport may undergo severe modification during *Botrytis* infection. In *Arabidopsis*, the pathogen was shown to highjack host sugar efflux systems by the direct induction of genes that encode SWEET transporters such as *At-SWEET4*, *At-SWEET7* and *At-SWEET15*, thus promoting pathogenesis (Chen *et al.*, 2010; Oliva and Quibod, 2017). However, genes that encode *At-STP4* and *At-STP13* sugar transporter proteins, causing sugar influx, may also be induced and play a role in plant defence (Fotopoulos *et al.*, 2003; Lemonnier *et al.*, 2014). The struggle for available sugars may involve the induction of cell wall invertases as well. Although the activity of invertase can be controlled at the post-translational level via their reaction products or inhibitory proteins (Fotopoulos, 2005), higher transcriptional activity of invertase genes was reported after a *Botrytis* infection. This was the case, for example, for *Sl-LIN6* in tomato (Berger *et al.*, 2004) and *At-βfruct1* in *Arabidopsis* (Fotopoulos *et al.*, 2003). Moreover, hexokinases are known to play a major role in defence, presumably as glucose sensors and regulators of gene expression (Rojas *et al.*, 2014; Aguilera-Alvarado and Sanchez-Nieto, 2017). The overexpression of two *At-HXK* genes in *Arabidopsis* increased resistance to necrotrophic fungi (Sarowar *et al.*, 2008).

The inhibition of photosynthetic mechanisms, along with the modulation of the enzymes implicated in carbohydrate metabolism, may lead to a modification of sugar contents in infected tissues. In *Botrytis*-infected tomato leaves, sugar levels decreased with an increase in the hexose-to-sucrose ratio (Berger *et al*., 2004, 2007; Camañes *et al.*, 2015). However, in grape berries the establishment of a pathogen-induced sink was reported, with an observed increase in fructose and glucose contents (Agudelo-Romero *et al.*, 2015). The balance between host and pathogen sugar utilization may define the outcome of the interaction. The manipulation of constitutive sugar equilibria through variations of host nitrogen nutrition in lettuce (Lecompte *et al.*, 2013) and tomato (Lecompte *et al.*, 2017) showed that the concentrations of sucrose and hexose relative to total sugar content were good markers of the plant’s susceptibility to *B. cinerea* and lesion growth a few days after inoculation. Moreover, the differentiated course of glucose and fructose contents in tomato stems after infection suggested that fructose played a specific role in the defence process.

In the present work, we used the nitrogen-dependent variation of tomato stem susceptibility to *B. cinerea* to examine the transcriptional activity of selected enzymes – invertases, sucrose synthases, hexokinases, fructokinases and phosphofructokinases – before and throughout the course of infection.

## MATERIALS AND METHODS

### Plant production and nitrate treatments

Protocols similar to those previously described (Lecompte *et al*., 2010, 2017) were used in this experiment. Plants were grown in a glasshouse from February to March 2018. Tomato seeds (*Solanum lycopersicum* ‘Clodano’; Syngenta, Wilmington, DE, USA) were sown and the seedlings were transferred 10 d after germination onto 7 × 7 × 6 cm rock wool blocks. After three additional weeks, the plants (bearing three or four leaves) were placed on top of 2-L pots filled with a mixture (1:1 v/v) of vermiculite and pozzolana. From germination to the beginning of the nitrate treatment, the plants were fertigated twice a day with a standard commercial nutrient solution (Liquoplant Rose; Plantin, Courthézon, France). The nitrate treatments were started 7 weeks after germination. Four nutrient solutions were used, containing nitrate concentrations of 0.5, 2, 10 and 20 mm. It had been previously shown in similar experiments that 10 mm is the optimal concentration for growth, and that 20 mm corresponds to an excess of nitrogen supply, while 0.5 and 2 mm correspond to severe and mild nitrogen stress, respectively. In the solutions with lower nitrate concentrations, the equilibrium in electric charges was maintained by replacing nitrates (monovalent anions) with sulphates (divalent anions), up to 7.5 mm SO_4_^2−^ in the solution containing the lowest nitrate concentration (0.5 mm NO_3_^−^). This substitution is unlikely to have affected plant growth since tomato is known not to be affected by very high sulphate supply concentrations (Lopez *et al.*, 1996). The concentration of other major nutrient elements was kept constant, at the following levels: 10 mm K^+^, 3.5 mm Mg^2+^, 3.25 mm Ca^2+^ and 0.02 mm HPO_4_^2−^. Micronutrients were present at the following concentrations (in µm): 0.5 B, 0.02 Cu^2+^, 8.2 Fe^2+^, 0.5 Mn^2+^, 0.01 MoO_4_^2−^ and 0.1 Zn^2+^. The plants were fertigated using a drip irrigation system (one dripper per pot) up to nine times a day depending on the climatic demand, with 2-min pulses. All plants received the same amount of water. The pH was adjusted to 6.0 in each treatment by adding H_3_PO_4_.

### *Inoculation of leaf pruning wounds with conidia of* B. cinerea

*Botrytis cinerea* strain BC1 was grown on plates containing potato dextrose agar medium (PDA; Difco, Detroit, MI, USA) in a growth chamber (18 °C night, 22 °C day and 14 h daylight). Conidia were collected in sterile ultra-pure water from the surface of 14-d-old cultures. Each suspension was filtered through a 30-µm mesh sterile filter to remove mycelium fragments, and adjusted to a concentration of 10^7^ conidia mL^−1^ with the help of a haemocytometer.

Thirty-five plants were used for each nitrate treatment, corresponding to seven batches of five biological replicates, sampled just before inoculation [0 d post-inoculation (DPI)], and at 2, 4 and 7 DPI (one batch of mock-inoculated and one batch of *Botrytis*-inoculated plants at each date). Inoculations were carried out in the morning (between 0900 and 1000 h, local time) on the petiole stubs (5–7 mm long) that remained on the stems after excision of the sixth leaf. The wounds received a 10-µL aliquot of either the spore suspension (*Botrytis*-inoculated plants) or sterile water (mock-inoculated plants).

To foster disease development, the *Botrytis*- and mock-inoculated plants were placed in a randomized design in two growth chambers set at 21 °C day/18 °C night, 90 % relative humidity and 13 h of daylight [200 µmol m^−2^ s^−1^ photosynthetic photon fluence rate (PPFR)]. During this period, the plants were fertigated manually twice a day until drainage, thus maintaining the nitrate treatments until the end of the experiment.

The disease was assessed by measuring the length of the lesion expanding on the stems on a daily basis. The area under the disease progress curve (AUDPC) was computed as: AUDPC = [(*Y*_1_/2) + Σ _2_^*n* − 1^ (*Y*_*j*_ + (*Y*_*n*_/2))][*I*], where *Y*_*j*_ was the observed lesion (in mm) at the *j*th observation time, *n* was the total number of observations, and *I* was the interval between each observation (in days, equal to 1 in our case).

### Sample collection

Fragments of symptomless stem, 2 cm long, were collected on *Botrytis*-infected plants on each side of the lesions, ~1 cm from the lesion margins, at 2, 4 and 7 DPI. Similar positions on the stem (between the fifth and sixth internode) were sampled from intact plants at 0 DPI and from mock-inoculated plants at 2, 4 and 7 DPI. The collected samples were immediately frozen in liquid nitrogen. Frozen stem tissues were ground into fine powder with liquid nitrogen using a mixer mill (MM301; Retsh, Haan, Germany) at a frequency of 30 Hz for 30 s. Powders were stored in airtight pillboxes at −80 °C until analysis.

The absence of RNA of fungal origin in the collected stem samples was verified by RT-qPCR as described below using a standard curve of *Botrytis* mRNA dilution in 100 ng µL^−1^ of tomato mRNA. The α-tubulin gene was used for quantification (Wu *et al.*, 2017).

### Biochemical analyses of plant tissue

Soluble sugars (glucose, fructose and sucrose) were extracted from 10 mg of powder using a 1-mL methanol/water mixture (1:1 v/v) and 300 µL of chloroform under continuous agitation. After centrifugation, 800 µL of the supernatant was evaporated under vacuum at room temperature. After the addition of 1.6 mL of ultrapure water and 10 mg of PVPP, the extracts were vortexed, centrifuged and purified using a C-18 cartridge (Sep-Pak; Waters, Milford, MA, USA) and a 0.2-µm filter (Pall, NY, USA). The sugars were determined by HPLC using a Sugar-Pak II pre-column and a Sugar-Pak I 300 × 6.5 mm column (Waters, Milford, MA, USA). Separation was carried out at 85 °C and a flow rate of 0.6 mL min^−1^ using 50 mg L^−1^ EDTA-Na_2_Ca as eluent. The sugars were detected by measuring the refractive index with an RI Detector 410 (Waters, Milford, MA, USA), using Chromelon software (ThermoFisher Scientific, Waltham, MA, USA). Sugar contents were expressed in grams per gram of dry weight, and relative glucose, fructose and sucrose contents (RGC, RFC and RSC, respectively) were calculated as the ratio of glucose, fructose and sucrose, respectively, to the total content of all three.

### Gene expression analyses

The sequences of tomato genes involved in sucrose cleavage and sugar phosphorylation were obtained from GenBank (www.ncbi.nlm.nih.gov/genbank). A total of 31 genes were identified in the tomato genome (Supplementary Data Table S1). Regarding phosphofructokinases, only predicted sequences were available, which were used to identify the corresponding genes in the tomato genome database using BLAST analyses (*Solanaceae* Genomics Network, www.solgenomics.net; Tomato Genome version SL4.0 and Annotation ITAG4.0). In order to verify their putative annotation, a protein sequence alignment was performed with the protein sequences of phosphofructokinase genes identified in other plants (Mustroph *et al.*, 2007), using the ClustalW method with default parameters, and a phylogenetic tree was constructed (Supplementary Data Fig. S1). It showed that all putative phosphofructokinases seemed valid and relevant to use.

Total RNA was isolated from 200 mg of frozen stem powder using the commercial RNeasy Plant Mini Kit (Qiagen, Hilden, Germany). To avoid DNA contamination, a DNase treatment was carried out using the RNase-Free DNase set (Qiagen, Hilden, Germany) as described by the manufacturer. Total RNA was quantified by spectrophotometry and RNA integrity quality was verified by 1 % agarose gel electrophoresis.

Gene expressions were assessed by RT-qPCR. Total RNA (200 ng) was used for first-strand cDNA synthesis, using oligo-(dT)_15_ primers and the Reverse Transcriptase Core Kit (Eurogentec, Liège, Belgium) according to the manufacturer’s instructions. qPCR amplification was performed in a 384-well plate using a CFX384 cycler (Bio-Rad, Hercules, CA, USA). The Takyon No Rox SYBRCore Kit blue dTTP (Eurogentec, Liège, Belgium) was used with 1 µL of cDNA and 250 nm of forward and reverse primers and the following programme was applied: 4 min at 95 °C followed by 60 cycles of 10 s at 95 °C and 1 min at 60 °C. To confirm the specificity of the amplicon, each qPCR programme was followed by a melting curve assay. The specific primers of targeted genes are listed in Supplementary Data Table S1. Each reaction was carried out in an independent technical triplicate and included a no-template control and a reagent-only control. Quantitation cycles (C_q_) were defined based on the mean of the three replicates. The geometric mean of the expression level of actin (*ACT*) and cyclophilin (*CYP*) genes was used as an internal control (reference) to normalize the expression data for the target genes. The relative expression levels of target genes were calculated as fold changes in expression using the 2^−ΔΔCq^ method: $2^{-[(C_{q(target,sample)}-C_{q(reference,sample)})-(mean(C_{q(target,calibrator)}-C_{q(reference,calibrator)}))]}$ (Livak and Schmittgen, 2001), where the mean *C*q values corresponded to the mean of the five biological replicates. Results were log_2_-transformed. To assess the effect of nitrate treatments before inoculation, gene expressions at 2, 10 and 20 mm NO_3_^−^ were related to gene expressions at 0.5 mm NO_3_^−^. To assess the effect of infection, the gene expressions of *Botrytis*-inoculated plants were related to gene expressions of mock-inoculated plants on the same day under the respective nitrate treatments. Finally, to evaluate the change in gene expression over time, the gene expressions at 2, 4 and 7 d after inoculation were related to gene expressions before inoculation under the respective nitrate treatments.

### Statistical analyses

The data presented are the averages of five biological replicates (each in technical triplicates) ± standard error. The differences between treatments were analysed using a Kruskal–Wallis test followed by a Wilcoxon rank-sum test when necessary. All the statistical analyses were performed using R software.

## RESULTS

### Sufficient and over-optimal nitrate treatment increased fructose content and carbohydrate metabolism gene expressions before inoculation

**Fig. 1.**
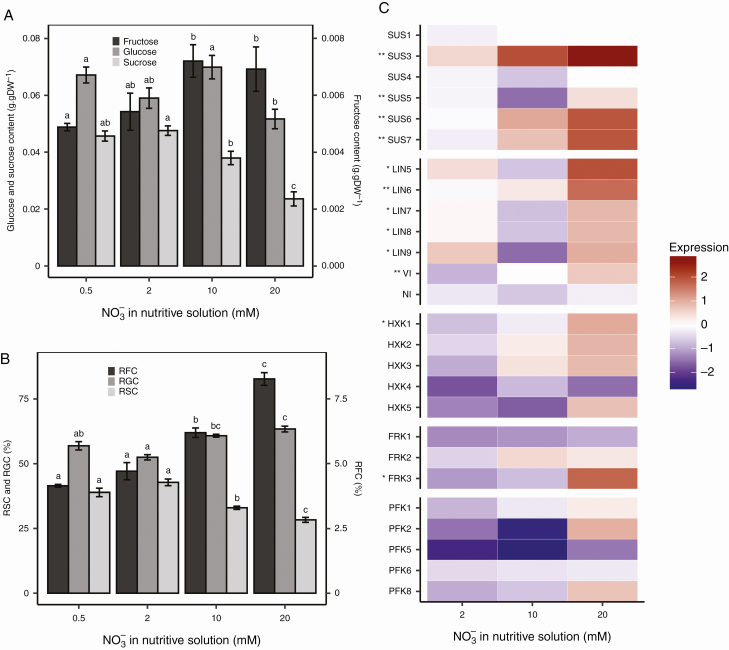
Carbohydrate metabolism status of tomato stems according to nitrate treatment prior to inoculation. Sugar contents (A) and relative sugar contents (RSC, RGC and RFC for sucrose, glucose and fructose, respectively) (B) before inoculation are plotted according to nitrate treatment. Each bar is the mean ± standard error of five observations, corresponding to five plants per nitrate treatment, one observation per plant. Different letters above columns indicate significant differences between nitrate treatments according to a Wilcoxon rank-sum test, one test per sugar. Relative gene expression (C) was calculated using the log_2_(2^−ΔΔ*C*q^) formula with *Sl-ACT* and *Sl-CYP* as a control for normalization. The mean of five observations (corresponding to five plants per nitrate treatment, one observation per plant) was computed. Asterisks next to gene names indicate level of significance according to nitrate treatment: **0.001 < *P* < 0.01, *0.01 < *P* < 0.05 (Kruskal–Wallis test).

Nitrogen availability affected the stem sugar contents before inoculation. The sucrose contents were higher, 0.046–0.048 g g^−1^ DW at 0.5 and 2 mm NO_3_^−^, compared with 0.038 g g^−1^ DW at 10 mm NO_3_^−^ and 0.024 g g^−1^ DW at 20 mm NO_3_^−^. The glucose contents were around 0.062 g g^−1^ DW regardless of the nitrate treatment. The fructose contents were slightly lower, 0.0049–0.0054 g g^−1^ DW at 0.5 and 2 mm NO_3_^−^, compared with 0.0069–0.0072 g g^−1^ DW at 10 and 20 mm NO_3_^−^ (Fig. 1A). For all nitrate treatments, the fructose contents were much lower than the sucrose and glucose contents. However, the relative fructose content (as defined in the Materials and methods section) increased with nitrogen availability, in parallel with a decrease in the relative sucrose content (Fig. 1B).

As the hexose-to-sucrose ratio was affected by nitrate treatments, we investigated the expression of genes that encode invertase, sucrose synthase, hexokinase, fructokinase and phosphofructokinase (Fig. 1C). Five among the 31 genes identified, namely *Sl-HXK6*, *Sl-FRK4*, *Sl-PFK3*, *Sl-PFK4* and *Sl-PFK7*, were not expressed in the stems regardless of the condition. All invertase and sucrose synthase genes were found to be expressed in the stems. Before inoculation, most of the genes that encode sucrose cleavage enzymes (invertase and sucrose synthase) showed significantly different expression levels depending on the nitrate treatment, except for *Sl-SUS1*, *Sl-SUS4* and *Sl-NI* (Fig. 1C). Most of the relative expressions were higher at 10 and 20 mm NO_3_^−^. However, a threshold of ±1.5 is usually recognized as the limit for the identification of differentiated expression, when using the log_2_(2^−ΔΔ*C*q^) relative value. Among all the genes examined, only the expression of *Sl-SUS3* at 20 mm NO_3_^−^ reached a significantly higher mean value than this 1.5 threshold. As for the kinase-encoding genes, a similar trend of higher relative expression for plants grown at 10 and 20 mm NO_3_^−^ was noted, but the differences were only significant for *Sl-HXK1* and *Sl-FRK3*.

### Disease severity decreased with high relative fructose content before inoculation

**Fig. 2.**
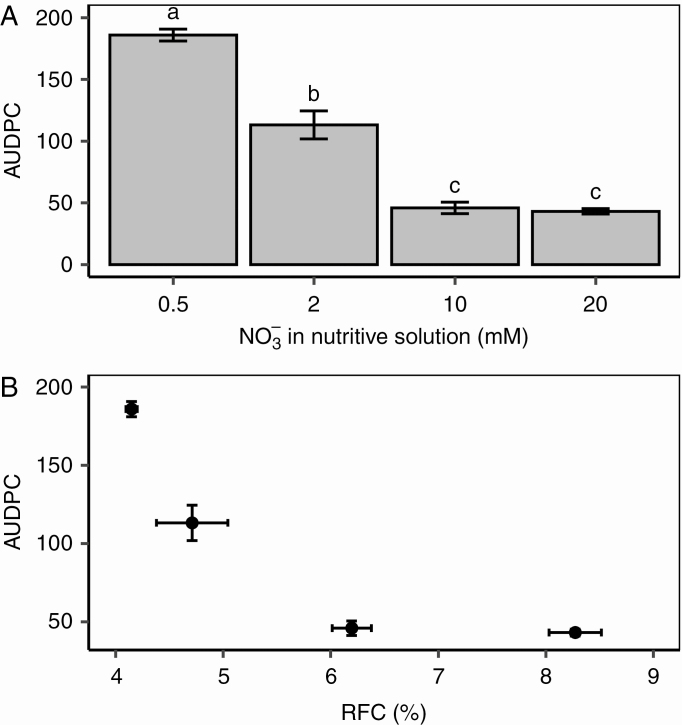
Relationship between disease severity, nitrate treatment and carbohydrate metabolism. (A) Average values of area under the disease progress curve (AUDPC) on tomato stems under four nitrate treatments. Each bar is the mean ± standard error of five observations (corresponding to five plants per nitrate treatment, one observation per plant). Different letters above columns indicate significant differences between nitrate treatments according to a Wilcoxon rank-sum test. (B) Relationship between the AUDPC and relative fructose content (RFC) of the tomato stem before inoculation. Each symbol corresponds to the mean ± standard error of five observations (corresponding to five plants per nitrate treatment, one observation per plant).

Four weeks after the beginning of the nitrate treatments, the plants were inoculated with *B. cinerea* and incubated for 7 d. The petiole stubs were colonized and lesions developed on the stems within 72–96 h. The AUDPC was computed based on the measurement of lesion lengths between 4 and 7 DPI. As expected based on previous studies, nitrate treatments affected plant susceptibility (*P* = 0.0003, Fig. 2A) with a >3-fold difference in AUDPC for plants grown with 0.5 mm NO_3_^−^ in the nutrient solution and those grown with 10 mm NO_3_^−^, and no difference between plants grown with 10 and 20 mm NO_3_^−^. We observed a tight curvilinear relationship between RFC before inoculation and AUDPC (Fig. 2B), while no significant results could be obtained when trying to fit various functions into the relationship between RSC and AUDPC, RGC and AUDPC, or plant nitrate content and AUDPC.

### Susceptible plants accumulated fructose after the onset of the infection under low nitrate treatment

**Fig. 3.**
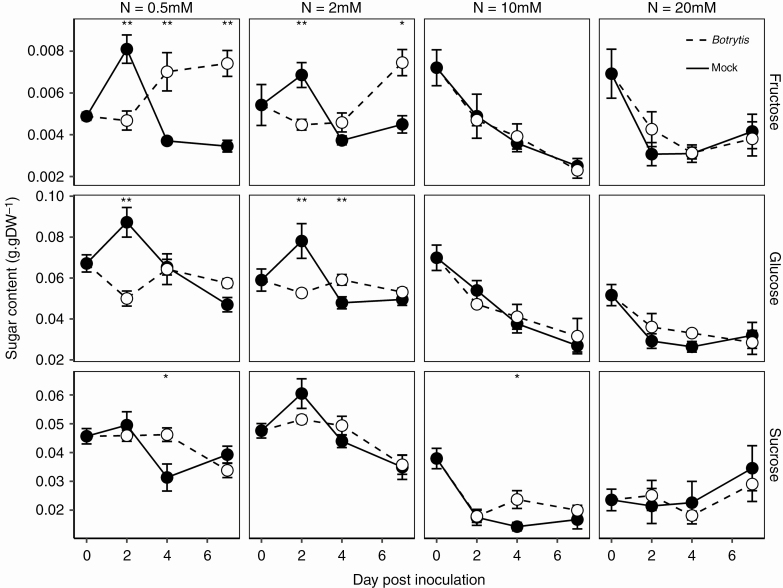
Course of sugar contents after *Botrytis* inoculation in fungus-free tomato stems. Each symbol is the mean ± standard error of five observations (corresponding to five plants per nitrate treatment, one observation per plant). Asterisks indicate levels of significance of the difference in *Botrytis*-inoculated plants compared with mock-inoculated plants with the same combination of nitrate treatment × day of measurement: **0.001 < *P* < 0.01, *0.01 < *P* < 0.05 (Kruskal–Wallis test).—

To ensure favourable incubation and regular disease development after leaf pruning, plants were transferred from a glasshouse to growth chambers, with adjustment of the air temperature, its relative humidity and the light intensity. Consequently, sugar metabolism was affected both in *Botrytis*-inoculated and mock-inoculated plants after this transfer (Fig. 3).

The sugar contents were analysed after confirmation by qPCR of the absence of *Botrytis* in asymptomatic regions of stems surrounding the lesions. We observed that nitrate treatments affected soluble sugar contents at all sampling dates after inoculation (Fig. 3). Under sufficient and over-optimal nitrate treatments (10 and 20 mm NO_3_^−^), no differences between *Botrytis* and mock-inoculated plants were observed. Fructose and glucose contents decreased between 0 and 7 DPI. Sucrose content dropped between 0 and 2 DPI, and remained roughly constant thereafter at 10 mm NO_3_^−^, while at 20 mm NO_3_^−^ it remained constant between 0 and 4 DPI and slightly increased between 4 and 7 DPI. Contrasts between *Botrytis*-inoculated and mock-inoculated plants were much more pronounced under low nitrate treatment. Fructose and glucose contents initially decreased after inoculation with *Botrytis*, but while glucose showed no significant change between 2 and 7 DPI, fructose started to accumulate between 2 and 4 DPI at 0.5 mm NO_3_^−^, and 4 DPI at 2 mm NO_3_^−^. An opposite pattern was observed in mock-inoculated plants, with an initial rise in glucose and fructose contents, followed by a decrease at 2 DPI. The sucrose content of infected plants started to drop at 4 DPI, while that of mock-inoculated plants started to decrease earlier, at 2 DPI. Thus, the major difference observed between infected and mock-inoculated plants was the accumulation of fructose in infected plants in the low nitrate treatments.

### Botrytis *infection affected the expression of genes that encode sucrose cleavage enzymes*

**Fig. 4.**
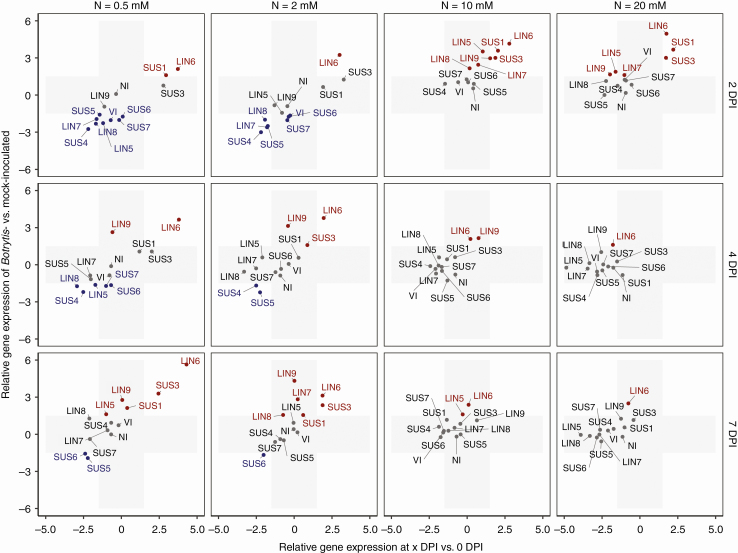
Changes in expression of genes that encode sucrose cleavage enzymes after *Botrytis* inoculation. The *x*-axis represents the fold change in gene expression of *Botrytis*-inoculated plants at 2, 4 or 7 DPI compared with plants before inoculation. The *y*-axis represents the fold change in gene expression of *Botrytis*-inoculated plants compared with mock-inoculated plants with the same combination of nitrate treatment × day of measurement. The grey area represents zones under the threshold level of significance, fixed at a 1.5-fold change. Genes in grey type show no difference in expression between *Botrytis*- and mock-inoculated plants, genes in red type are overexpressed and genes in blue type are underexpressed.

**Fig. 5.**
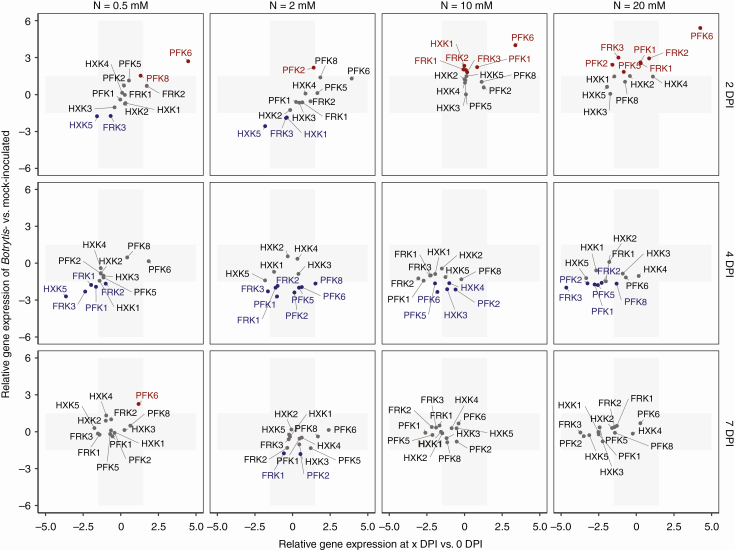
Changes in expression of hexose kinase genes during *Botrytis* infection. The *x*-axis represents the fold change in gene expression of *Botrytis*-inoculated plants at 2, 4 or 7 DPI compared with plants before inoculation. The *y*-axis represents the fold change in gene expression of *Botrytis*-inoculated plants compared with mock-inoculated plants with the same combination of nitrate treatment × day of measurement. The grey area represents zones under the threshold level of significance, fixed at a 1.5-fold change. Genes in grey type show no difference in expression between *Botrytis*- and mock-inoculated plants, genes in red type are overexpressed and genes in blue type are underexpressed.

The expression of genes involved in sugar metabolism after inoculation was examined. The expressions of sugar metabolism genes in *Botrytis*-inoculated plants relative to those of mock-inoculated plants measured on the same day (*y* axis) as well as the gene expressions of inoculated plants relative to those measured before inoculation (*x* axis) are plotted in Fig. 4 for sucrose-cleaving enzymes. The gene expressions that exceed the ± 1.5 threshold of ΔΔ*C*q are highlighted in Figs 4 and 5. Graphs that compare individual gene expressions under the four nitrate treatments and the related statistics are provided in Supplementary Data Fig. S2.

The nitrate treatments strongly affected gene expressions after inoculation, especially at 2 DPI (Supplementary Data Fig. S2). Two groups of sucrose synthases may be distinguished. On the one hand, *Botrytis* inoculation stimulated the expression of *Sl-SUS1* and *Sl-SUS3* at 2 DPI, but the transcript levels (relative to those of mock-inoculated plants) were much higher for plants grown at 10 and 20 mm NO_3_^−^ than for plants grown at lower nitrogen levels (Fig. 4, Supplementary Data Fig. S2A). When related to their levels of expression before inoculation, *Sl-SUS1* and *Sl-SUS3* were stimulated by the infection under all nitrate treatments, but more strongly under high nitrate treatment (Fig. 4). On the other hand, a second group of transcripts, corresponding to *Sl-SUS4* to *Sl-SUS7*, were lower at 0.5 and 2 mm NO_3_^−^ relative to mock-inoculated plants, whereas no such repression was detected at higher NO_3_^−^ concentrations. A similar contrast was observed for invertases. The relative expressions of *Sl-LIN5*, *Sl-LIN7*, *Sl-LIN8*, *Sl-LIN9* and *Sl-VI* were negative at 0.5 and 2 mm NO_3_^−^ and positive at 10 and 20 mm NO_3_^−^ (Fig. 4, Supplementary Data Fig. S2A). *Sl-LIN6* showed a particular pattern, since its relative expression was high regardless of the nitrate treatment, though it increased from 2.5 at 0.5 mm NO_3_^−^ to 5 at 20 mm NO_3_^−^. The gene that encoded the Sl-NI mitochondrial invertase hardly showed any variation after inoculation, regardless of the nitrate treatment. The observed contrasts in relative expression according to nitrate treatment were attenuated at 4 and 7 DPI (Supplementary Data Fig. S2B, C). Relative to mock-inoculated plants, *Sl-SUS1* and *Sl-SUS3* transcripts were higher under low nitrate treatments, especially at 7 DPI, while *Sl-SUS4* to *Sl-SUS7* were still repressed at 0.5 mm NO_3_^−^, and to a lesser extent at 2 mm NO_3_^−^ (Supplementary Data Fig. S2C). The *Sl-LIN5*, *Sl-LIN7*, *Sl-LIN8* and *Sl-VI* invertases were still repressed, in comparison with mock-inoculated plants, at 4 DPI, but not at 7 DPI. By contrast, *Sl-LIN6* and *Sl-LIN9* were strongly expressed at 4 and 7 DPI, with higher transcript levels under low nitrate treatments. To summarize, *Sl-SUS1* and *Sl-SUS3* were activated upon infection and the early activation of these genes, as well as those that encode cell wall invertases, was associated with reduced susceptibility, while on the contrary the early repression of *Sl-SUS4* to *Sl-SUS7* and cell wall invertases, except for *Sl-LIN6*, was associated with increased susceptibility.

### Botrytis *infection affected hexose kinase gene expressions*

Similarly to genes that encode sucrose cleavage enzymes, several genes that encode hexose kinases showed contrasted expressions 2 d after inoculation, depending on the nitrate treatment. *Sl-HXK1*, *Sl-FRK1* to *Sl-FRK3*, *Sl-PFK1* and to a lesser extent *Sl-HXK2* and *Sl-HXK5* showed enhanced expression at 10 and 20 mm NO_3_^−^ relative to mock-inoculated plants (Fig. 5, Supplementary Data Fig. S2A). In contrast, *Sl-HXK5* and *Sl-FRK3* were repressed at 0.5 and 2 mm NO_3_^−^. The putative phosphofructokinase gene *Sl-PFK6* was strongly activated under all nitrate treatments relative to the expression in mock-inoculated plants and relative to its expression level before inoculation. Differences between nitrate treatments were much less pronounced at 4 DPI (Supplementary Data Fig. S2B), at which time the transcript levels of *Sl-HXK5*, *Sl-FRK1* to *Sl-FRK3*, *Sl-PFK1*, *Sl-PFK2*, *Sl-PFK5*, *Sl-PFK6* and *Sl-PFK8* were lower in *Botrytis*-inoculated plants in comparison with mock-inoculated ones regardless of the nitrate treatments. At 7 DPI, the relative expression of hexose kinases was very low, regardless of the nitrate treatments. Thus, the condition of reduced susceptibility was associated with activation of all fructokinase genes, along with *Sl-HXK1*, *Sl-HXK2* and *Sl-HXK5* hexokinases and the *Sl-PFK1* phosphofructokinase. Conversely, the condition of enhanced susceptibility was associated with repression of *Sl-HXK5* and *Sl-FRK3*.

## DISCUSSION

In the present work, we used contrasted nitrate treatments to examine the sugar metabolism associated with the increased resistance or susceptibility of tomato stems to *B. cinerea*. We analysed the content of soluble sugars (sucrose, glucose and fructose) and the expression of genes involved in the catabolism of sucrose and the first phosphorylating steps of glycolysis. The metabolites and transcript levels were quantified in symptomless stem tissues surrounding *Botrytis* lesions. Stems are sink tissues for which little is known regarding the relationship between sugars and the response to infection. The particularity of sink tissues lies in the activity of sucrose synthase, generally not detected in source tissues (Wind *et al.*, 2010). Among the 31 genes for invertases, sucrose synthases, hexokinases, fructokinases and phosphofructokinases analysed in this work (Supplementary Data Table S1), we detected the expression of 26 genes. All sucrose synthase and invertase genes are expressed in tomato stems, *Sl-SUS1*, *Sl-SUS3*, *Sl-SUS5*, *Sl-LIN8* and *Sl-VI* being the most abundant isoforms, according to Hyun *et al.* (2011) and Qin *et al.* (2016). Fructokinase genes *Sl-FRK1* to *Sl-FRK3* are expressed in all vascular tissues, unlike *Sl-FRK4*, which is specific to anthers and pollen (German *et al.*, 2002; Granot *et al.*, 2013; Stein *et al.*, 2018) and accordingly was not detected in this study. We did not record transcripts of the *Sl-HXK6* hexokinase gene and of the *Sl-PFK3*, *Sl-PFK4* and *Sl-PFK7* putative phosphofructokinase genes; it can be hypothesized that these genes are not expressed in tomato stems. The main objective of gene expression analysis was to compare the transcriptional activity of wounded and mock-inoculated plants with wounded and *Botrytis*-inoculated plants, under various nitrate treatments. Knowing that the expression levels of the genes studied are not quantitatively linked to their enzymatic activity (Keurentjes *et al.*, 2008) and that the metabolic transformation of sugars relies on multiple reversible reactions, the relationship between gene expression and sugar content observed in this work may only indicate a qualitative defence response induced by the various nitrate treatments. Moreover, a leaf was cut off in order to carry out both the mock inoculation and the *Botrytis* inoculation. In a study on *Arabidopsis*, a modification in the expression of the *AtC/VIF2* gene, which encodes a cell wall invertase inhibitor, was measured with a mock-inoculation corresponding to an infiltration of 10 mM MgCl_2_ (Bonfig *et al.*, 2010). This may indicate the possible effect of wounding on invertase activity unrelated to the pathogen infection. For all these reasons, the gene expression levels of *Botrytis*-inoculated plants presented here were expressed relative to those of mock-inoculated plants.

We initially analysed the effect of nitrate treatments on constitutive gene expressions and sugar contents before inoculation. These treatments mostly affected sucrose content, which was twice as high at 0.5 and 2 mm NO_3_^−^ compared with 20 mm NO_3_^−^. In the tomato cultivar used in this work, the fructose content was much lower than the glucose content, and slightly lower under low nitrate treatments. Other studies have described higher sucrose and total reducing sugar contents in sink tissues under nitrogen stress (Cazetta *et al.*, 1999; Krapp *et al.*, 2011). Most probably, sucrose (and starch) accumulation may be linked to reduced growth at low nitrogen levels. Accordingly, in this study we found that several, but not all, isoforms of cell-wall invertases and sucrose synthases showed reduced expression levels under low nitrate treatments. A reduced activity of invertases at low nitrogen availability has been shown in cassava leaves (Cruz *et al.*, 2003) and maize kernels (Cazetta *et al.*, 1999).

Many reports, regularly reviewed in the past decade, have emphasized the role of host sugars and related enzymes in plant–pathogen interactions (Bolton, 2009; Bolouri Moghaddam and Van den Ende, 2012; Tauzin and Giardina, 2014; Kanwar and Jha, 2019). The plant’s nitrogen status is known to affect disease incidence and development in multiple ways (Fagard *et al.*, 2014), and nitrogen nutrition has already proved to be a modulator of *Botrytis* susceptibility in tomato leaves and fruit (Vega *et al.*, 2015) as well as stems (Lecompte *et al.*, 2017). Plant infection by pathogens is known to induce a massive reprogramming of the host cell machinery from growth to defence. This takes place via the inhibition of photosynthesis at infection sites in photosynthetic tissues, and the coincident activation of sugar transporters and sucrose-cleaving enzymes, the modulation of carbohydrate metabolism and hexose-to-sucrose ratio, and the production of end products of hexose metabolism, e.g. cell wall compounds (Bellincampi *et al.*, 2014), hormones (Beyers *et al.*, 2014) and antimicrobial compounds (Tiku, 2018). It has been suggested that high levels of soluble sugars in the host generally favour plant resistance to pathogens (Morkunas and Ratajczak, 2014). Accordingly, in one study by Hoffland *et al.* (1999) tomato leaf susceptibility to *B. cinerea* was positively correlated with the soluble carbohydrate content. However, this was not the case in lettuce leaves (Lecompte *et al.*, 2013), and tomato stems, as shown in the present study and in previous work (Lecompte *et al.*, 2017). The effect of elevated nitrate treatments on reduced tomato stem susceptibility to *B. cinerea* is not correlated, whether positively or negatively, with the total content of soluble sugars. Instead, a strong and robust correlation between stem susceptibility and the constitutive relative fructose content has been shown in previous studies (Lecompte *et al.*, 2017) and in the present one. In this study we have observed an early accumulation of hexoses in mock-inoculated plants grown under low nitrate treatments. By contrast, a rise in fructose content was observed later (between 2 and 4 DPI) in infected plants under low nitrate treatments. Regarding mock-inoculated plants, the observed hexose accumulation may have been due to leaf pruning and the environmental changes associated with the transfer of plants into the growth chamber. However, nitrogen nutrition interfered with the response, since no hexose accumulation was observed under high nitrate treatments. Regarding *Botrytis*-inoculated plants, under low nitrate treatments the late accumulation of fructose might be related to the lower expression of hexokinases and fructokinases observed. Thus, under low nitrate treatments less fructose might be involved in the glycolysis and further synthesis of defence compounds. However, since the measurements were performed in tissues neighbouring the lesions, it could also be hypothesized that hexoses were not used in these asymptomatic cells but rather remobilized in sucrose and then transported to the infection site. Studying the host sugar dynamics in tissues colonized with fungal mycelium is complex, since the retrieval of host sugars by the pathogen must be estimated using specific labelling techniques (Dulermo *et al.*, 2009).

In the present work, the inoculation of *B. cinerea* triggered high and early transcription of all isoforms of invertases, as well as *Sl-SUS1* and *Sl-SUS3*, under the nitrate treatments that provided 10 and 20 mm NO_3_^−^ in the nutrient solution, and this transcriptional activity was associated with phenotypes with reduced susceptibility. By contrast, more susceptible plants displayed repressed expressions of *Sl-SUS4* to *Sl-SUS7* and of some invertase isoform genes under low nitrate treatments. Interestingly, nitrogen availability affected the constitutive expression of *Sl-SUS3* before and after infection, while the overexpression of *Sl-SUS1* was only observable after infection. *Sl-SUS3* expression in healthy tomato plants is mostly associated with vascular tissues, and changes according to stem maturity (Goren *et al.*, 2011). In our work, the overexpression, before infection, of *Sl-SUS3* at sufficient (10 mm) and over-optimal (20 mm) nitrogen availability could correspond to the higher stem volumes observed in these treatments. However, the overexpression of *Sl-SUS1* is dependent on both infection and high nitrogen availability. Here we highlight the conditional involvement of some specific isoforms of sucrose synthases in a successful defence.

The *Sl-LIN6* invertase was overexpressed in all nitrate treatments during the course of infection. In leaves of tomato and *Arabidopsis* infected by *B. cinerea*, *Sl-LIN6* (Berger *et al.*, 2004) and *At-CWIN1* (Veillet *et al.*, 2016) invertases are activated. In addition, increased apoplastic hexose uptake and upregulation of several sugar transporter genes have been observed after infection by *B. cinerea* (Lemonnier *et al.*, 2014; Veillet *et al.*, 2017; Bezrutczyk *et al.*, 2018). The internalization of sugars may participate in ‘pathogen starvation’, a defence mechanism that potentially limits the availability of apoplastic sugar for the pathogen (Bezrutczyk *et al.*, 2018). However, in this work increased expression of genes that encode sucrose synthases, which have a cytosolic activity, was observed after inoculation. This strengthens the hypothesis of higher recruitment of hexoses by the host tissues. Oppositely, the downregulation of some invertases in susceptible plants may facilitate the maintenance of elevated apoplastic sucrose availability, in favour of the pathogen.

Less susceptible plants observed in this work upregulated the transcription of sugar metabolism genes 2 d after inoculation. Several studies have already highlighted the importance of the early-stage response for increased resistance (Hyun *et al.*, 2011; Birkenbihl *et al.*, 2012; De Cremer *et al.*, 2013). In this work, susceptible plants showed delayed expressions of *Sl-SUS1*, *Sl-SUS3*, *Sl-LIN8* and *Sl-LIN9* along with the continuous repression of several *Sl-SUS4* to *Sl-SUS7* isoforms throughout the course of infection. This repression of sucrose cleavage enzymes was associated with poorly effective defence. It originated from the low nitrogen status of the host, but whether the fungus is able to manipulate this expression in a nitrogen-dependent manner remains to be examined.

Hexokinases are known to be involved in plant–pathogen interaction (Morkunas and Ratajczak, 2014; Rojas *et al.*, 2014), but fructokinase and phosphofructokinase gene expression after infection by a necrotrophic fungus has not been described thoroughly in the literature. Here, more susceptible plants showed repressed fructokinase transcript levels in comparison with mock-inoculated plants, while more resistant plants under sufficient and over-optimal nitrate treatments showed higher levels of transcripts of *Sl-FRK1* to *Sl-FRK3* early after inoculation. In accordance with lower fructokinase transcript levels, higher levels of fructose content were observed at low nitrogen availability, thus suggesting lower mobilization of fructose for glycolysis. A similar accumulation of fructose in tomato stems after infection at low nitrogen availability has already been observed in another tomato cultivar (Lecompte *et al.*, 2017).

Recent work on metabolomics and transcriptomics highlighted cell wall reinforcement with callose, and lignins, as a successful strategy against *B. cinerea* (Asselbergh *et al.*, 2007; Curvers *et al.*, 2010; Yang *et al.*, 2018). Sucrose synthase cleaves sucrose into fructose and UDP-glucose, the latter being presumed to trigger cell wall reinforcement. The early expression of fructokinase genes in resistant plants observed in this study suggests that the activity of sucrose synthase may not be limited to cell wall reinforcement, unless fructose catabolism in glycolysis is a fortuitous consequence of defence activation. However, other work suggests that glycolysis and subsequent pathways are useful for defence (Agudelo-Romero *et al.*, 2015; Camañes *et al.*, 2015; AbuQamar *et al.*, 2016). On the one hand, GS/GOGAT (glutamine synthetase/glutamine oxaloglutarate aminotransferase) activity and the assimilation of nitrogen may control, in addition to sugar regulation, the maintenance of cell homeostasis against pathogenic fungi (Seifi *et al.*, 2013). On the other hand, the pentose phosphate pathway supports the synthesis of precursors of phenylpropanoid compounds that are synthesized in response to *B. cinerea* (Ma *et al.*, 2018). Thus, supplementary fructose synthesis from sucrose synthase activity, followed by its phosphorylation and entry into glycolysis, may be needed for increased resistance in sink tissues.

In conclusion, this study shows that the increased resistance of the tomato stem to *B. cinerea* is supported by early activation of genes that encode sucrose cleavage enzymes: invertases and sucrose synthases. This is only observed in conditions of sufficient or over-optimal nitrogen availability. By contrast, in conditions of low nitrogen availability early repression of several sucrose synthase and invertase genes is observed after infection. The cytosolic activity of sucrose synthase associated with the enhanced expression of hexokinases and fructokinases in resistant plants supports the hypothesis of increased synthesis of cell wall components and/or the recruitment of additional fructose in glycolysis and subsequent pathways for an effective defence.

## SUPPLEMENTARY DATA

Supplementary data are available online at https://academic.oup.com/aob and consist of the following. Table S1: sequence of *Solanum lycopersicum* primers used in this study. Figure S1: phylogenetic tree of hexose kinases in the tomato and phosphofructokinases in plants. Figure S2: relative expression of carbohydrate metabolism genes at 2 (A), 4 (B) and 7 (C) DPI.

mcaa155_suppl_Supplementary_Material
