# Supplementary material for: Regulation of sugar metabolism genes in the nitrogen-dependent susceptibility of tomato stems to Botrytis cinerea
Source: Ann Bot. 2020 Aug 27;127(1):143–54. doi: 10.1093/aob/mcaa155 (PMC7750717; doi:10.1093/aob/mcaa155)

**Table S1: Sequence of *Solanum lycopersicum*** primers used in this study

| **Gene** | **Accession no.** | **Locus Symbol** | **Forward (5’ ⭢ 3’)** | **Reverse (5’ ⭢ 3’)** | **Prod. Length (pb)** |
| --- | --- | --- | --- | --- | --- |
| ACT | NM_001321306.1 | Solyc03g078400 | TGTCCCTATTTACGAGGGTTATGC | AGTTAAATCACGACCAGCAAGAT | 73 |
| CyP | NM_001247559.1 | Solyc01g111170 | CGTCGTGTTTGGACAAGTTG | CCGCAGTCAGCAATAACCA | 108 |
| SUS1 | NM_001247726.2 | Solyc12g009300 | CTGCTGAGTGAATGAAGGTC | GATACTAAATGGAAATGAAACAC | 100 |
| SUS3 | NM_001313910.1 | Solyc07g042550 | GGTTTCTGTCTGATTGTTATCC | ACAGAAGGGAAAAATGGCAAA | 109 |
| SUS4 | NM_001331183.1 | Solyc09g098590 | AACGTGGAGCATACCACTCT | CAGCATATTGGATCACTGATTTG | 183 |
| SUS5 | XM_004243632.4 | Solyc07g042520 | GGCCTAATTCATTTGGTAATTC | GTGCTGAAATTTCATCTGGAC | 169 |
| SUS6 | XM_004235340.4 | Solyc03g098290 | CTTGCCAGTTGCACCATTAG | GTAGCTTGCTTCGCGATTTC | 186 |
| SUS7 | XM_004232624.4 | Solyc02g081300 | CAGCAGTGGCTGTGTCTTTC | CCATGCTCGTGCAAGTAAAC | 103 |
| LIN5 | NM_001247864.1 | Solyc09g010080 | CTGAATGCTTGGAGCATGGAT | GAGGGATTTTTGTGAACATCATCTACTG | 140 |
| LIN6 | NM_001246913.2 | Solyc10g083290 | AGCACATTTATTCGCCTTCAACAA | CTTTGTGACGTGGCATAATAAGAT | 125 |
| LIN7 | NM_001247772.1 | Solyc09g010090 | GAACGGAGCCAATCACAATTG | TCCCCCTTTTACCATAGTTCCTT | 114 |
| LIN8 | NM_001324396.1 | Solyc10g083300 | AGTCATTGAATGCATGGAGCAT | GAGCAATCAAATACGTCACCACAA | 121 |
| LIN9 | NM_001247140.1 | Solyc08g079080 | ACTGGGTCAACCAACGAATC | TGCCCTCATACTTGATCCAT | 97 |
| VI | NM_001247914.2 | Solyc03g083910 | TCCTTCCCTTTGCAAGACTTGT | TCTCCCTCTTCCCTTTCTTGATG | 102 |
| NI | XM_010320928.3 | Solyc01g058010 | GCCTCTTGATGGAAGGAATGGT | CCAGAATCAACAGGTGCAACAC | 93 |
| HXK1 | NM_001247028.1 | Solyc03g121070 | TCGGCACCAGCTTTGATCC | CGACTCCCACCGTCACTTTC | 159 |
| HXK2 | NM_001247477.2 | Solyc06g066440 | TTGGCGCTGCACTCCTC | AGTTCCATATAATTTCTTTGCACCCTTT | 223 |
| HXK3 | NM_001247781.2 | Solyc12g008510 | TGGGGAGTGGGGAAAAGTTG | ACGCTTTTCTTTCCCACCCA | 268 |
| HXK4 | NM_001247788.2 | Solyc04g081400 | TGTAGCAATGGATGGAGGCTT | GCCTCTATAAATCCAATCATCAATCCT | 295 |
| HXK5 | XM_004231915.4 | Solyc02g091830 | AGTGGGGACTCATCCTTGTG | TCGTTTACTGCTTGTTTGCGT | 217 |
| HXK6 | XM_010314953.3 | Solyc11g065220 | TACAAACACCACCGCCCTTT | AGCTCACTGTTTTCCTTCTCCTC | 214 |
| FRK1 | NM_001246964.2 | Solyc03g006860 | TCTGGCATCTGTTGGTCGGT | CTACCCTATTTTTCCCTGCATGTCTC | 76 |
| FRK2 | NM_001246959.2 | Solyc06g073190 | GCAATCCCAGCTTTGCCTAC | TGATGATGTAAAGGAGAAGTTACAAGG | 254 |
| FRK3 | NM_001247467.2 | Solyc02g091490 | GTCGTCGTCGGCTAGTCTTT | GGGAAAATAGTAGCAGCGGAGA | 95 |
| FRK4 | NM_001247277.2 | Solyc10g017620 | CATGGAAGAGTGAGTGGCGT | TATCCACATGATTTTGTTACAGGGATAG | 368 |
| PFK1 | XM_004235135.4 | Solyc03g093520 | ATTCACCTTCCGCAGAGCA | TGATCCAAGGGTTGAGCGATT | 109 |
| PFK2 | XM_004236651.4 | Solyc04g014270 | ACGTGTCTCCGATCTCTCCA | GCGGCAGCATTTTTCGGTT | 250 |
| PFK3 | XM_004236594.4 | Solyc04g015200 | GGGTTCGTTTGTGGACCGA | GAGGAGGACAGAGAGAAGCCA | 191 |
| PFK4 | XM_004238461.3 | Solyc04g072580 | TTCCATGGGATCATTGGTTGATAGTT | CTAGGAATGTAATCAGACAAATGAGGAAC | 117 |
| PFK5 | XM_004243552.4 | Solyc07g045160 | GAGGCTTATGTCCCGGTCTC | TGTTCCACCGCGTTTATGGA | 176 |
| PFK6 | XM_004245088.4 | Solyc08g066100 | CATAAGCGACAGCACTCACAAAG | AGCAACACCTCCACAACATAGAA | 229 |
| PFK7 | XM_004250088.4 | Solyc11g010450 | GTCAGATCCTCCAAACGCACA | CGTATTATATGAGTGGTATTTATTCTTCGTCAA | 188 |
| PFK8 | XM_004253054.4 | Solyc12g095880 | CCAGATGAAGGTGGTGAAAGGAG | GAAATGCACCCCTCTGGGAC | 208 |

**Fig. S1: Phylogenetic tree of hexose kinases in the tomato and phosphofructokinases in plants.** Sequence similarities of tomato protein sequences and 34 phosphofructokinase protein sequences from different plants were calculated using ClustalW method with default parameters.


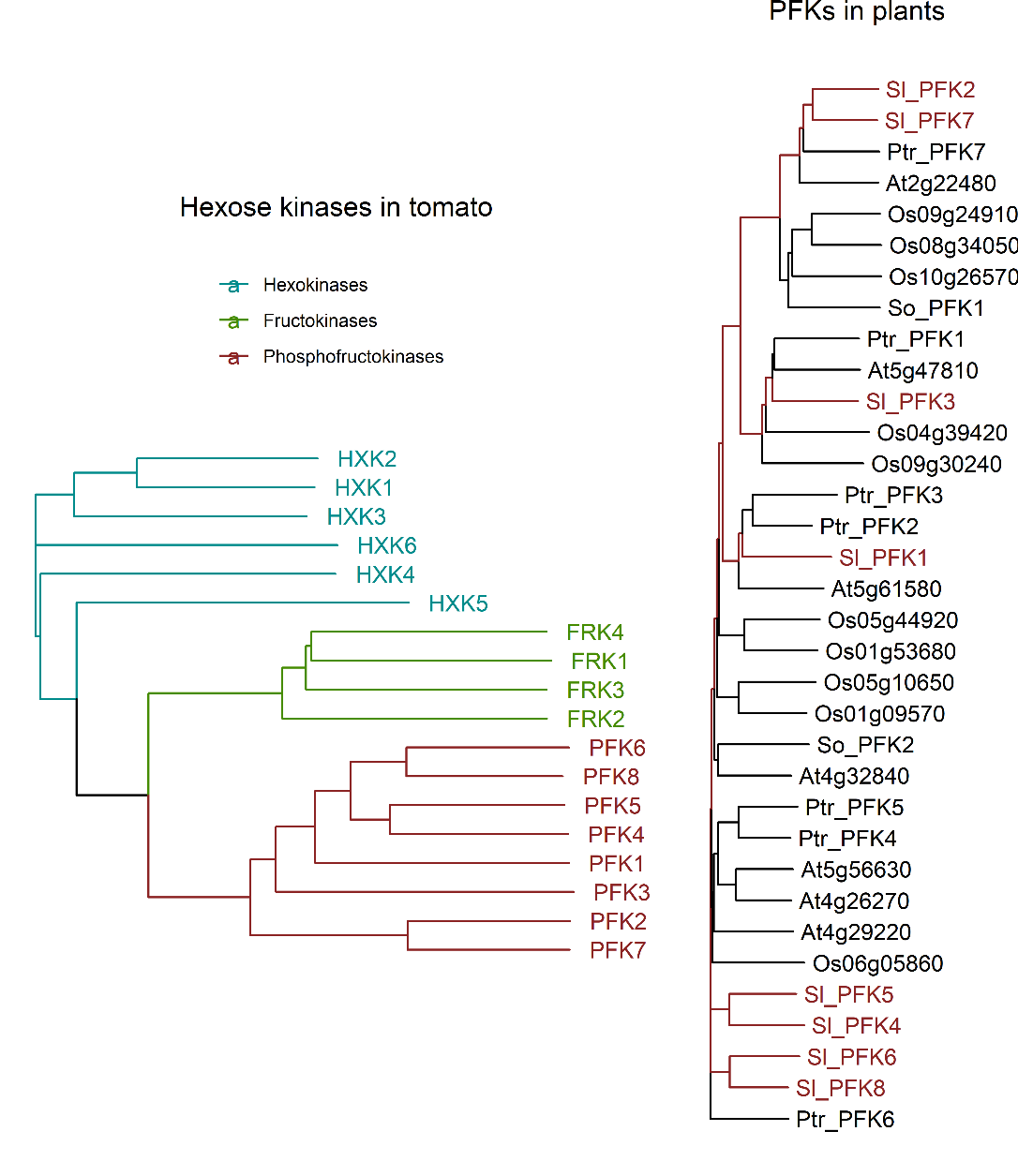


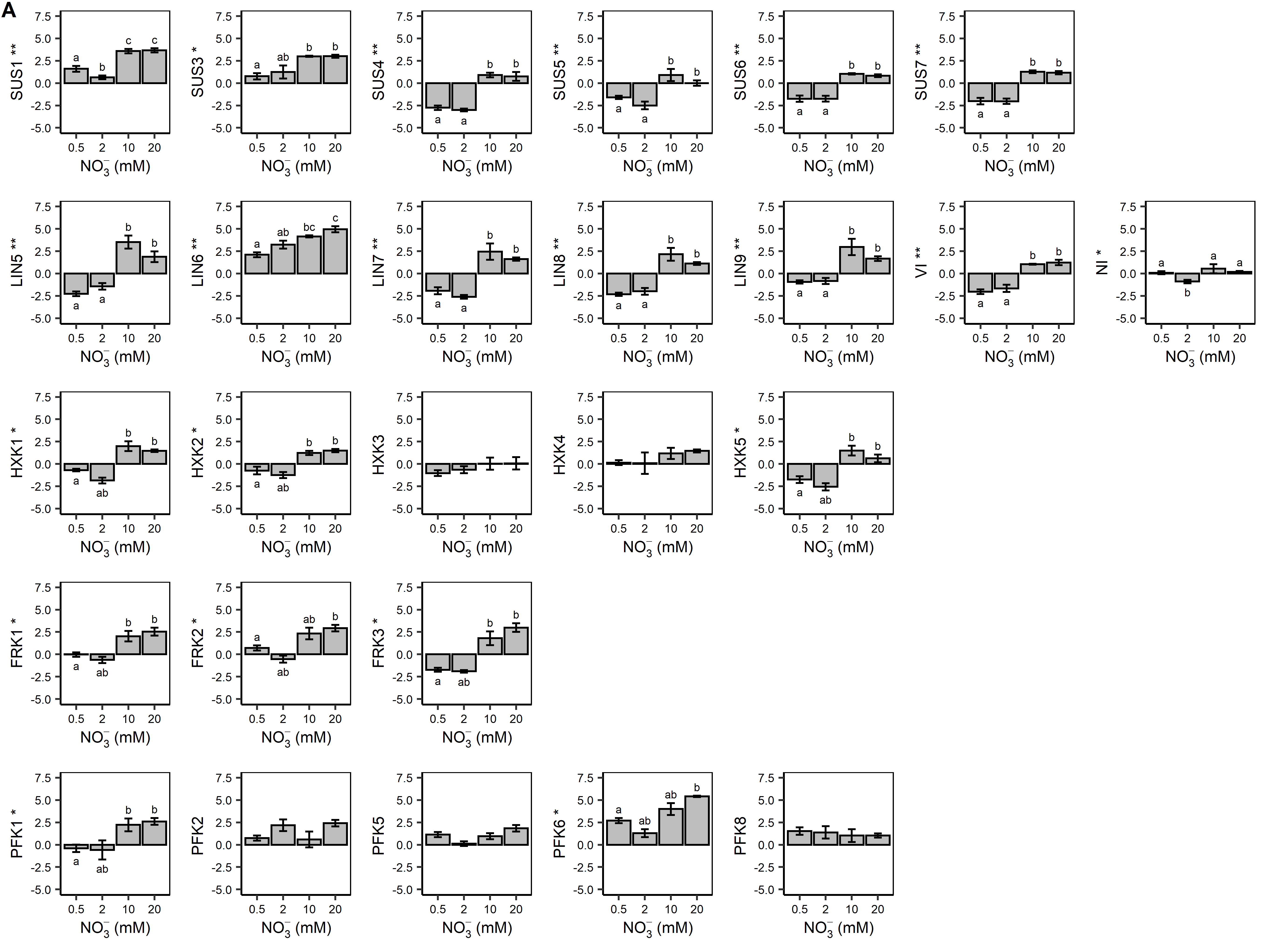
**Fig. S2: Relative expression of carbohydrate metabolism genes at 2 (A), 4 (B) and 7 (C) days post inoculation** Each bar is the mean ± standard error of five observations (corresponding to five plants per nitrate level, one observation per plant). Asterisks beside gene names indicate the levels of significance according to N-treatments (Kruskal-Wallis test, *** when P < 0.001, ** when 0.001 < P < 0.01 and * when 0.01 < P < 0.05). Letters indicate significant differences between N-treatments according to a Wilcoxon rank-sum test, one test per gene.


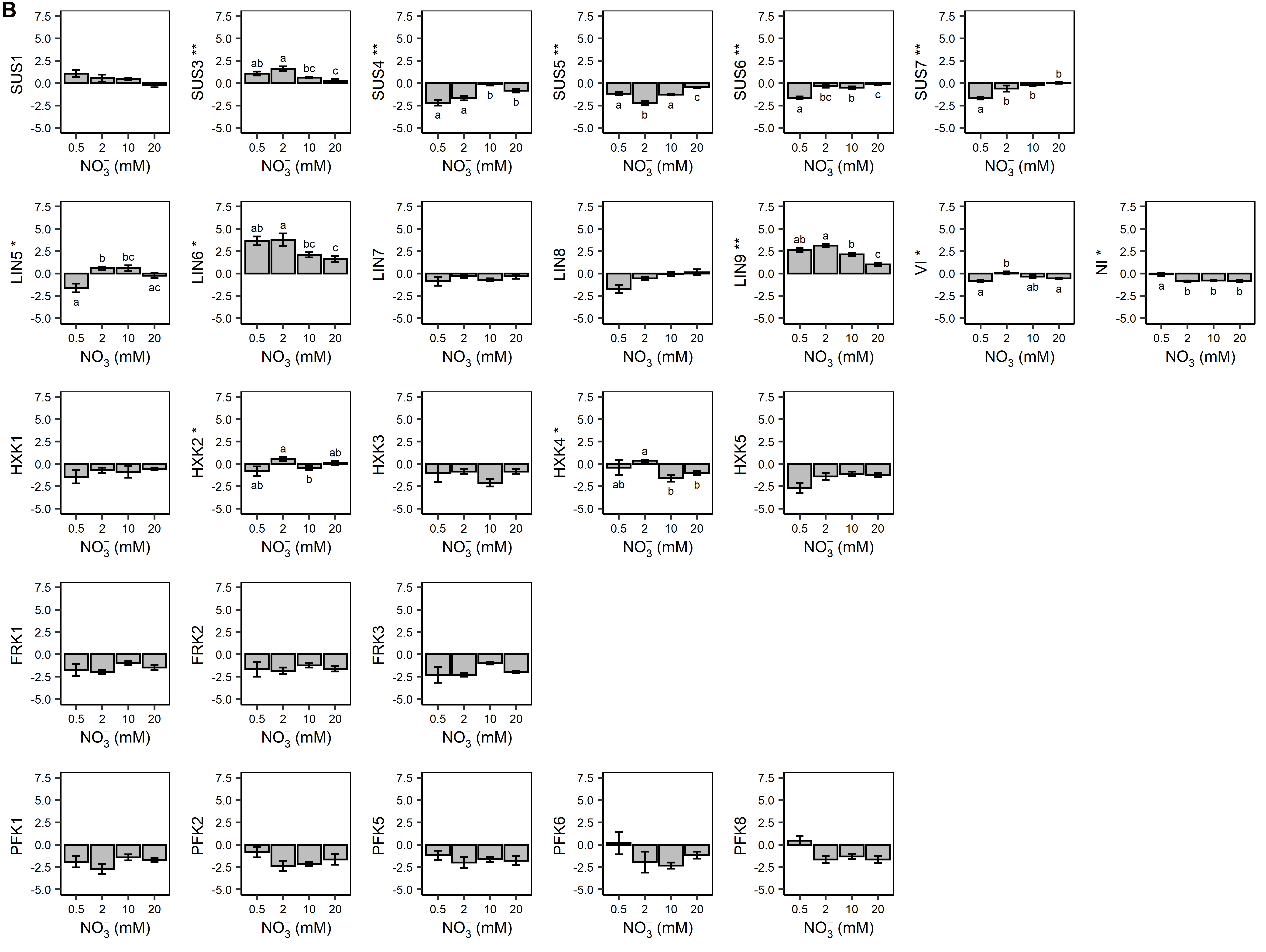


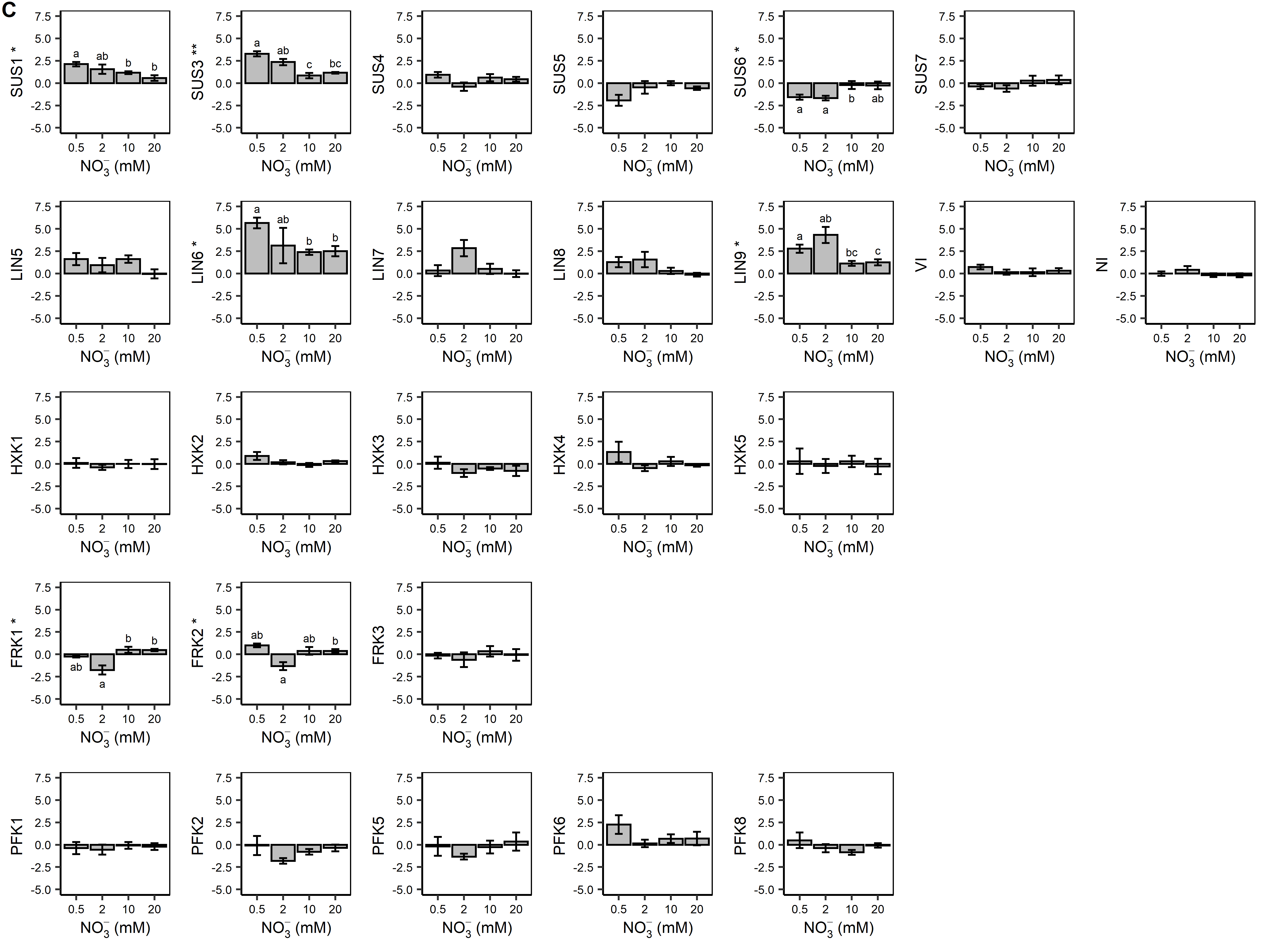

Supplement: mcaa155_suppl_Supplementary_Material [file mcaa155_suppl_supplementary_material.doc]
